# Quantifying the carbon footprint of conference travel: the case of NMR meetings

**DOI:** 10.5194/mr-6-243-2025

**Published:** 2025-11-10

**Authors:** Lucky N. Kapoor, Natália Ružičková, Predrag Živadinović, Valentin Leitner, Maria Anna Sisak, Cecelia Mweka, Jeroen Dobbelaere, Georgios Katsaros, Paul Schanda

**Affiliations:** 1 Institute of Science and Technology Austria, Am Campus 1, 3400 Klosterneuburg, Austria

## Abstract

Conference travel contributes to the climate footprint of academic research. Here, we provide a quantitative estimate of the carbon emissions associated with conference attendance by analyzing travel data from participants of 10 international conferences in the field of magnetic resonance, namely EUROMAR, ENC and ICMRBS. We find that attending a EUROMAR conference produces, on average, more than 1 t ${\mathrm{CO}}_{2\;\mathrm{eq}.}$ . For the analyzed conferences outside Europe, the corresponding value is about 2–3 times higher, on average, with intercontinental trips amounting to up to 5 t. We compare these conference-related emissions to other activities associated with research and show that conference travel is a substantial portion of the total climate footprint of a researcher in magnetic resonance. We explore several strategies to reduce these emissions, including the impact of selecting conference venues more strategically and the possibility of decentralized conferences. Through a detailed comparison of train versus air travel – accounting for both direct and infrastructure-related emissions – we demonstrate that train travel offers considerable carbon savings. These data may provide a basis for strategic choices of future conferences in the field and for individuals deciding on their conference attendance.

## 1 Introduction

Reducing the emissions of greenhouse gases is an important challenge to limit global warming [Bibr bib1.bibx24]. Despite the increasing awareness, global annual greenhouse gas (GHG) emissions have continued to increase steadily, reaching approximately 59±6.6 Gt ${\mathrm{CO}}_{2\;\mathrm{eq}.}$ in 2023, which is 62 % higher than in 1990 [Bibr bib1.bibx20].

Academic research activity also leads to carbon emissions, and factors such as the production of research consumables (e.g.,  chemicals), the construction and maintenance of scientific instrumentation and buildings, commuting to the workplace, and conference travel have been identified as the activities with the largest footprint [Bibr bib1.bibx8].

The climate crisis is a direct consequence of the quantity of greenhouse gases emitted, and it is, thus, a fundamentally *quantitative* matter; naturally, the analysis of causes and possible solutions shall therefore use a quantitative approach. Any meaningful action to mitigate the climate crisis – be it at the level of individuals, organizations, communities or countries – must be grounded in accurate data: deciding in which fields to make changes requires identifying which of our activities are the largest contributors to our carbon emissions.

According to the International Energy Agency (IEA), the transport sector is responsible for around one-fifth of all human-made global GHG emissions.In the following, we will use the term ${\mathrm{CO}}_{2\;\mathrm{eq}.}$ , in which gases other than ${\mathrm{CO}}_{2}$ are considered, too, taking into account their global warming potential. Air travel is estimated to contribute around 2.5 % of the global ${\mathrm{CO}}_{2\;\mathrm{eq}.}$ emissions, accounting for around $1\times 10^{9}$ t (2021) [Bibr bib1.bibx2]. The climate impact of aviation extends beyond the directly emitted ${\mathrm{CO}}_{2}$ . When accounting for non- ${\mathrm{CO}}_{2}$ effects, such as nitrogen oxide emissions, water vapor and contrail formation at high altitudes, the sector's overall contribution to global warming is estimated to be around 3.5 % to 4 % [Bibr bib1.bibx28].

Given these numbers, one may argue that air travel contributes only little to the overall ${\mathrm{CO}}_{2\;\mathrm{eq}.}$ emissions and that removing flights would not solve the climate crisis. However, considering that approximately 90 % of the world's population does not fly [Bibr bib1.bibx14], the contribution of air travel to the carbon footprint of those who do fly can be substantial. As an example, an out-and-back transatlantic trip emits approximately 4.5 t of ${\mathrm{CO}}_{2\;\mathrm{eq}.}$ . To put this number into perspective, the International Panel on Climate Change's Special Report (SR15) estimated that the remaining global carbon budget for a 66 % chance of limiting warming to 1.5 °C is approximately 420 Gt ${\mathrm{CO}}_{2\;\mathrm{eq}.}$ (status: 2017). Even 1.5 °C of global warming – which is predicted to be surpassed very quickly [Bibr bib1.bibx30] – is projected to result in substantial impacts on natural systems [Bibr bib1.bibx29]. With a world population of approximately 8 billion and applying distributive justice [Bibr bib1.bibx1], a per-person annual “budget” of approximately 4.5 t ${\mathrm{CO}}_{2\;\mathrm{eq}.}$ can be estimated for 2025. This number has to reach zero in 2050.

The current per capita ${\mathrm{CO}}_{2\;\mathrm{eq}.}$ emissions are unevenly distributed, with approximately 14 t ${\mathrm{CO}}_{2\;\mathrm{eq}.}$ (USA, Australia) or 7 t ${\mathrm{CO}}_{2\;\mathrm{eq}.}$ (Germany) [Bibr bib1.bibx36], i.e., far above the annual per capita budget of 4.5 t. In light of these numbers, it is clear that a transatlantic trip to a conference (of the order of 4–5 t ${\mathrm{CO}}_{2\;\mathrm{eq}.}$ ) is far from negligible and cannot be aligned with a fair distribution of emission rights and limiting damage to humanity and natural systems.

Several studies have analyzed the carbon footprint of academic research in general and of travel in particular, e.g., [Bibr bib1.bibx8], [Bibr bib1.bibx3] and [Bibr bib1.bibx13]. In this spirit, we decided to analyze the emissions related to magnetic-resonance (MR) conferences. These conferences are driven by the community, and as a community, we can consider options to reduce their climate impact. We have compiled participant lists from 10 major MR meetings over the last 10 years, extracted the presumed travel trajectories of the participants and converted these to carbon emissions. To perform this conversion realistically and accurately, we have also reviewed the conversion factors, including indirect emissions associated with, e.g., railway infrastructure. We compare the average per-person emissions of conference attendance to other research-related GHG emissions of a typical magnetic-resonance laboratory.

In our search for potential avenues to reduce the carbon footprint of EUROMAR, we find that the choice of the conference location is an important factor in overall emissions, mirroring previous findings [Bibr bib1.bibx31]. We explored the possibility of having decentralized (two-site) conferences and found some potential (of the order of one-fourth) for reduction.

**Figure 1 F1:**
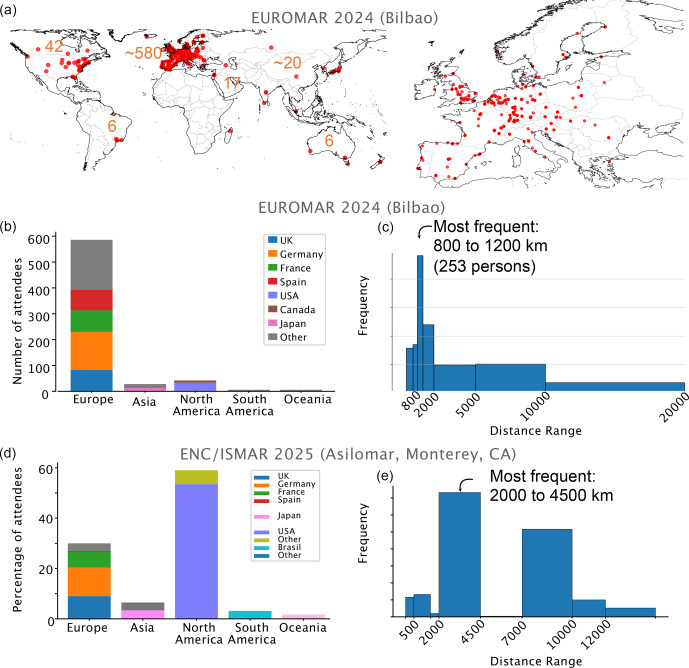
Distribution of participants at EUROMAR 2024 and ENC/ISMAR 2025 conferences. **(a–c)** EUROMAR 2024 (Bilbao), **(d–e)** ENC/ISMAR 2025 (Asilomar, California). Data were obtained by analyzing a list of participants with their affiliations, assuming that the city of the institute of affiliation is the origin of the travel to the conference.

## 2 Magnetic resonance conference travel in numbers

### 2.1 Distances traveled: past conferences

For our quantitative analysis of conference-travel-related emissions, we collected data for major MR conferences from 2016 to 2025 (see methods in the Appendix): EUROMAR editions 2016 (Aarhus), 2017 (Warsaw), 2018 (Nantes), 2019 (Berlin, joint with ISMAR), 2022 (Utrecht), 2023 (Glasgow) and 2024 (Bilbao); International Conference on Magnetic Resonance in Biological Systems (ICMRBS) editions 2022 (Boston) and 2024 (Seoul); and ENC-ISMAR 2025 (Asilomar, California). These conferences lasted between 4 and 6 d and hosted between approximately 470 (ICMRBS) and 1100 (joint EUROMAR-ISMAR) participants; most of the EUROMAR conferences hosted ca. 600–700 participants.

A notable first observation is the geographical distribution of attendees, as illustrated for the case of EUROMAR 2024 in Fig. [Fig F1]. The majority of participants at EUROMAR conferences, often exceeding 80 %, come from Europe. The conference location slightly alters the distribution: we systematically detected additional “local” participants, comprising about 20–30 participants affiliated to the institute of the organizers, as well as more participants from the hosting country. This trend is particularly pronounced for the 2024 ICMRBS in Seoul, e.g., where 200 of the ca. 580 participants were from the Republic of Korea (Fig. S1); for EUROMAR 2016 (Aarhus), 69 of the 620 were affiliated to a Danish institution, while this number was below 10 for all other EUROMAR editions. Likewise, ICMRBS 2022 (Boston) showed a strong attendance of participants from the Boston area (75 of ca. 470). This stronger inclusion of the local scientific community is, of course, a desired effect of moving the conference to different places. (We note that for ENC 2025 (Asilomar), this “local” effect is much less pronounced.)

Figure [Fig F2] shows cumulative distribution functions for all conferences (one-way distances). It illustrates that for conferences outside Europe, about half of the participants travel several thousand kilometers (round trip). Examples of travel-distance distributions are shown in Fig. [Fig F1]c for EUROMAR 2024 (Bilbao) and Fig. [Fig F1]e for ENC/ISMAR 2025 (California). At EUROMAR conferences, the most frequent distance traveled is between 800 and 1200 km, accounting for approximately 40 % of the participants. Another 25 % travel distances shorter than 800 km, and the remaining approx. 35 % travel distances longer than 2000 km, including long intra-European and overseas travel.

**Figure 2 F2:**
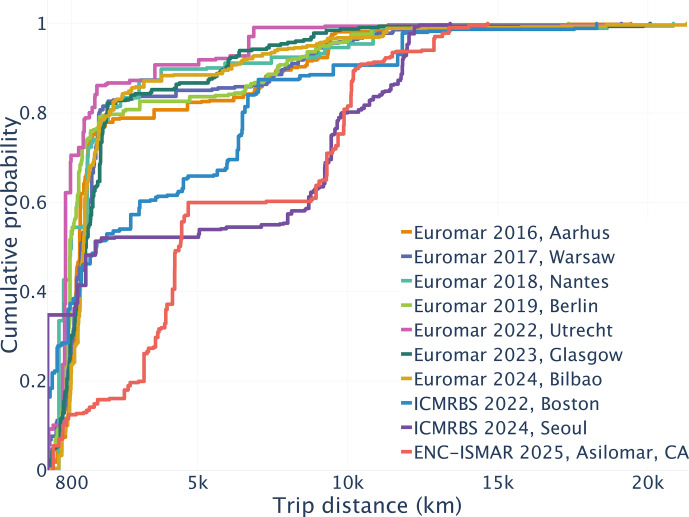
Distances traveled by conference participants shown as a cumulative distribution function, based on one-way distances. The trip distance is equal to the train distance if shorter than 800 km (i.e., the distance of train journeys is explicitly taken into account, using *Carbontracer* [Bibr bib1.bibx41] or to flight distance otherwise.

### Calculation of ${\mathrm{CO}}_{2\;\mathrm{eq}.}$ emissions

To translate this distance information into carbon emissions, assumptions must be made about the choice of transportation, as well as the respective per-kilometer emissions for trains and planes. (Note that we did not consider car travel for the European conferences.) Similarly to a study by [Bibr bib1.bibx25], we assumed that attendees traveling for 400 km or less (one-way) choose to travel by train, while for longer distances, air travel is chosen. We also repeated the calculation for a cutoff of 800 km (one-way). In doing so, we explicitly used the actual distance of the train trip as calculated by *Carbontracer* [Bibr bib1.bibx41]. The 400 km cutoff, used in the previous study [Bibr bib1.bibx25], is also supported by travel data from our institute: we reviewed several thousand trips of scientists traveling to/from our institute (data not shown), and found that a 400 km one-way distance is a reasonable cutoff above which air travel is chosen. For conferences outside Europe, we did not consider train travel as an option. For participants within 400 km of Asilomar or Boston, we assumed transport by car. We note that a more realistic metric would be to use travel times, rather than distances, as a criterion for travel preference. However, we refrained from such a metric because we lack the tools to calculate travel times efficiently for hundreds or even thousands of trips.

For the conversion from kilometers to metric tons of emitted ${\mathrm{CO}}_{2\;\mathrm{eq}.}$ , we conducted a literature survey, paying particular attention to using realistic values and including indirect emissions (see Appendix for details). In brief, for air travel, one needs to take into consideration (i) the fact that short distances consume more fuel per kilometer than long-distance flights, (ii) radiative forcing and (iii) emissions related to infrastructure (airports). Likewise, for train transportation, we sought to obtain a holistic picture that includes not only the emissions related to producing electricity for propelling the trains, but also indirect effects. In particular, the construction and maintenance of railway lines and buildings are significant factors in train travel. Details on how we converted distances to emitted carbon are provided in the Appendix. In brief, a value of 25 g of ${\mathrm{CO}}_{2\;\mathrm{eq}.}$ per passenger kilometer in addition to the direct emissions is a realistic estimate for European countries. The carbon emissions for the production of electricity vary significantly for different countries (from 8 g ${\mathrm{CO}}_{2\;\mathrm{eq}.}$ (kW h)^−1^ in Sweden to 594 g ${\mathrm{CO}}_{2\;\mathrm{eq}.}$ (kW h)^−1^ in Poland [Bibr bib1.bibx12]. We have considered these differences; see the Appendix.

Our calculations have several shortcomings, which we shall list here. First, we do not know the mode of transportation chosen by each attendee. Moreover, for flights, we assumed direct flights from the airport closest to the participant's affiliation to the conference location. In reality, many journeys include connecting flights, which can significantly increase the carbon footprint by up to 100 kg [Bibr bib1.bibx7]. Thus, the estimated emissions due to air travel are likely underestimated by approximately 20 %. We also ignored possible car travel, which would slightly reduce the emissions (compared to flights) or increase the emissions (compared to train travel). Our estimates of emissions related to train travel are on the “pessimistic” side, and many websites of railway companies report lower numbers, usually because the indirect emissions are omitted. We explicitly want to be conservative here and avoid greenwashing of trains (see also below). We also note that the choice between train and air travel will depend not only on distance but also on the train connections and the availability of night trains. Finally, we chose distance as the criterion in our analysis. We note that the time of travel can also be used, especially for conference locations where the variance in time of travel for a fixed distance is large (e.g., due to high-speed rails connecting some cities but not others). However, using time as a criterion has the disadvantage of excluding night trains, which for a given distance typically takes more time. Technically, it is more challenging to estimate the travel time accurately than to calculate the distance precisely, and we thus use distance as the criterion. We note that a survey conducted among conference participants would be helpful to generate more accurate data. Such a questionnaire could be part of the conference organizers' feedback collection process.

Figure [Fig F3] shows the total and per-participant travel-related emissions of the analyzed conferences. The total travel-related carbon emissions of EUROMAR conferences were of the order of 700–900 t total or about 1.2–1.3 t ${\mathrm{CO}}_{2}$ per participant (assuming that flights are taken for distances longer than 400 km) or approximately 1 t (assuming that trains are taken up to 800 km). The per-person carbon footprint of conferences outside Europe is about 2 to 3 times higher due to longer distances traveled and also the less widespread availability and use of train travel for long-distance travel in, e.g., the USA.

### 2.2 Travel-related emissions dominate the total conference-related emissions

In addition to travel, accommodation, catering and the conference site lead to further carbon emissions. Data about emissions of hotels have been collected, e.g., by the Cornell Hotel Sustainability Benchmarking (CHSB) Index [Bibr bib1.bibx35] or via the greenhouse gas conversion factors published annually by the UK Department for Environment, Food & Rural Affairs (DEFRA) or the French ADEME, and are available via web servers such as the hotel footprinting tool (https://www.hotelfootprints.org/, last access: 15 May 2025). The emissions scale roughly with the standing of the hotel due to the larger space for higher-rated hotels. For a 3-star hotel, they are in the range of 10–20 kg per night and person in a European country, which amounts to several tens of kilograms for an entire EUROMAR stay.

Meals can be estimated to produce approx. 5.6 kg ${\mathrm{CO}}_{2\;\mathrm{eq}.}$ for a meat-based meal, 3.8 kg ${\mathrm{CO}}_{2\;\mathrm{eq}.}$ for a vegetarian meal and 2.9 kg ${\mathrm{CO}}_{2\;\mathrm{eq}.}$ for a plant-based diet [Bibr bib1.bibx38], which sums to approx. 14–28 kg ${\mathrm{CO}}_{2\;\mathrm{eq}.}$ for a 5 d conference. One can certainly debate whether meals should be counted as conference-specific, as they replace the ones the participants would have consumed if they were not attending the meeting.

The conference venue requires electricity and possibly natural gas for heating. Although we do not have precise values for the venues of previous EUROMAR conferences, one can estimate the corresponding carbon footprint to be approx. 10 kg per participant for the entire conference [Bibr bib1.bibx10].

Overall, we estimate that the GHG emissions associated with conference attendance, excluding travel, amount to several tens of kilograms ${\mathrm{CO}}_{2\;\mathrm{eq}.}$ . Thus, transportation to the conference site, with typically more than 1 t of ${\mathrm{CO}}_{2\;\mathrm{eq}.}$ , is the main contributor to the overall footprint of conferences.

**Figure 3 F3:**
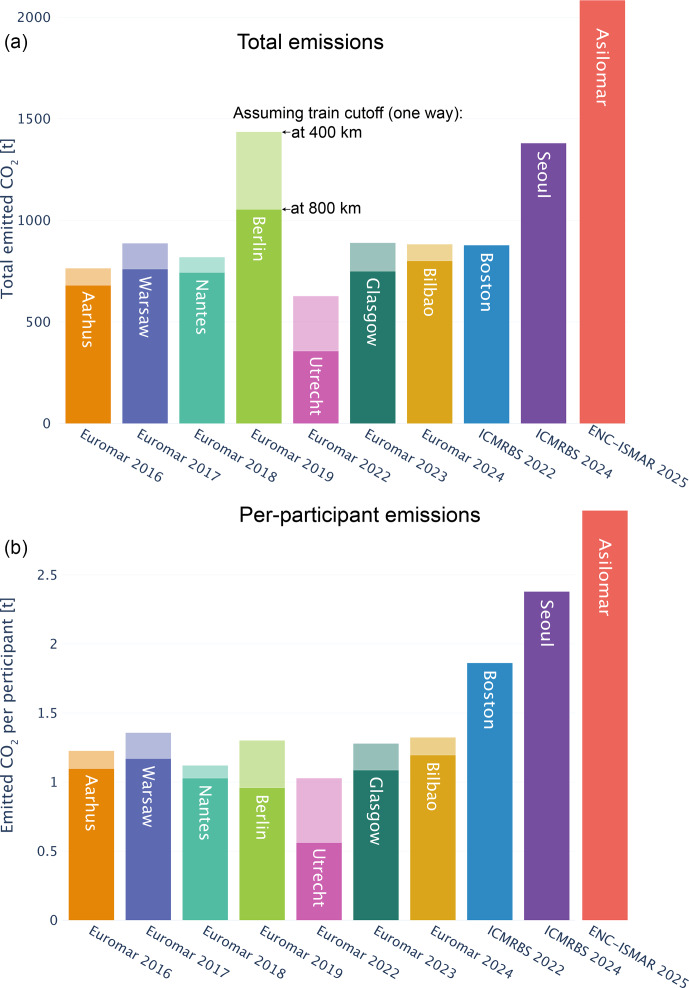
Travel-related ${\mathrm{CO}}_{2}$ emissions from major magnetic-resonance conferences. **(A)** Total emissions resulting from the conferences, assuming that participants used air travel if the distance exceeded either 800 km one-way (dark colors) or 400 km (light colors) and train travel otherwise (for EUROMAR conferences). In other words, the darker colors show the more sustainable scenario (people are willing to take trains up to 800 km and fly only distances exceeding 800 km), since more journeys are taken by train in this scenario. For conferences in Boston, Seoul and Monterey (Asilomar), car travel was assumed for distances below 400 km and air travel was assumed otherwise. The number of participants was estimated to be (from left to right) 700, 620, 650, 723, 1100, 635, 690, 670, 470 and 583. **(B)** Per-participant emissions. It is noteworthy that ICMRBS 2024 in Seoul had a particularly large share of local participants (200 out of 583; see Fig. S1), and the per-person average excluding local participants exceeded 3.1 t. Similarly, 76 out of the 470 delegates at the 2022 Boston edition were from Boston.

**Figure 4 F4:**
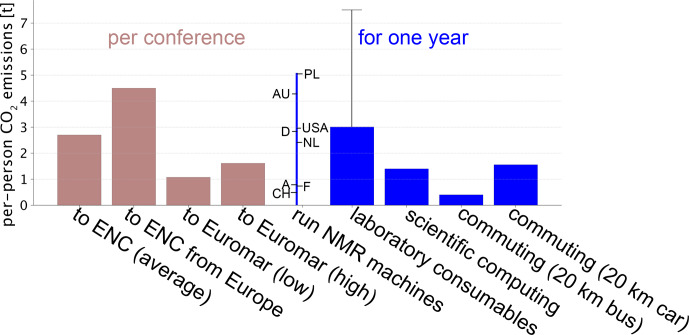
Comparison of per-person ${\mathrm{CO}}_{2}$ emissions from attending conferences compared to those due to activities directly related to research in a magnetic-resonance laboratory, all calculated as per-person emissions, as described in the main text. The emissions for operating NMR machines (three spectrometers used by 20 persons, 600 MHz with solution- and solid-state NMR, 700 MHz solids and 800 MHz with solid-state and cryoprobe solution-state NMR) were calculated for different countries (Poland, Australia, USA, Germany, Netherlands, Austria, France, Switzerland) and differ because of the different shares of fossil fuels for electricity production.

## 3 How do these numbers compare to our other research activities?

Our analysis shows that, on average, attending a typical magnetic-resonance conference produces travel-related carbon emissions ranging from approximately 1 to 3 t ${\mathrm{CO}}_{2}$ per person, where the variability is primarily due to the conference location. The travel-related carbon footprint of attending an overseas conference is approximately 4 to 5 t per participant. One way of putting these numbers into context is to compare them to the above-mentioned annual “carbon budget” of 4.5 t, which is not to be exceeded to limit global warming to 1.5 °C (although this is a number that must decrease over time).

Another interesting way to see these numbers is to compare them to the carbon emissions directly related to our actual research, i.e., everything needed to generate scientific data in the first place, before possibly presenting results at conferences. In light of those, are travel-related emissions possibly negligible anyhow?

For NMR laboratories, typical activities that generate carbon emissions are (i) the emissions due to production of NMR machines (supercooled magnets, electronics), (ii) the power consumption to operate these machines and to provide cryogenics, (iii) purchase of computers and running IT infrastructure (e.g., clusters), (iv) construction, maintenance and heating/cooling of the buildings we work in, (v) production of samples (e.g., (bio)chemistry laboratory, isotopes, solvents), and (vi) commuting to/from work.

For calculating points (i) and (ii), we used data from the R-NMR project online calculator, which considers the power consumption needed for running the console and possibly compressors/pumps, as well as power related to He and N_2_ boil-off and liquefaction (https://csdm.dk/rnmr/consumption.html, last access: 15 May 2025, version 1.1.5, created by Thomas Vosegaard). As an example, we presume a facility with three NMR systems at 600, 700 and 800 MHz (4 K magnets) with a solid-state probe and two cryoprobes. Assuming 20 group members use this infrastructure, the resulting carbon footprint per person ranges from 0.8 to 5 t yr^−1^, with variability related to the mode of electricity production and, thus, the country. In other words, participation in a 5 d EUROMAR conference has a larger impact than doing NMR for the entire year in, e.g., France. Figure [Fig F4] shows this model calculation for various countries.

Consumables required to produce samples have been identified as the main contributor to carbon emissions in research laboratories [Bibr bib1.bibx3]. In a study involving hundreds of laboratories in France, the emissions related to consumables (i.e., their production, transport and disposal) were estimated to account for approximately 2.7 to 3 t ${\mathrm{CO}}_{2\;\mathrm{eq}.}$ per person [Bibr bib1.bibx8]; for a study focusing on chemistry laboratories, a value of 2.3 t was reported [Bibr bib1.bibx11]. There is a large variability, with values up to approximately 7 t. We performed our own estimations at our institute, which covers a wide range of fields, using a cost-based conversion metric, and identified a value at the upper end of this range. Clearly, the exact type of research is a critical determinant of emissions, and these numbers – 2.5 to 7 t of ${\mathrm{CO}}_{2\;\mathrm{eq}.}$ emissions per researcher – serve as a rough estimate for comparison.

To quantitatively assess other contributors like computers/IT, buildings or commuting, we used data gathered by the ISTA's sustainability office related to our institute. ISTA is a growing institute on the outskirts of Vienna, and started from zero in 2009. It currently hosts approximately 85 research groups, has 1165 employees in total (700 researchers) and spans research in most fields of natural sciences, including experimental groups in physics, chemistry, biology and mathematics, theory, and machine learning. Its focus is research; teaching is limited to PhD student courses. Approximately 83 % of electric power in Austria is produced from renewable resources, which is relevant as the numbers vary for different countries.

At our institute, scientific computing is estimated to have a carbon footprint of approximately 7 % of the total ${\mathrm{CO}}_{2\;\mathrm{eq}.}$ emissions (power); another 0.7 % is related to the production of the IT hardware; computing/IT together amount to ca. 1.4 t ${\mathrm{CO}}_{2\;\mathrm{eq}.}$ per year per employee. Note that this number will be higher in a country with a more fossil-heavy energy mix, such as the USA or China (approximately 60 % fossil fuel share) or Germany (ca. 40 % fossil), and slightly lower for France ( <10 % fossil), compared to Austria (ca. 17 % fossil).

15 % of our carbon footprint is derived from the electricity we use (2.5 t) (excluding computing) and 4 % from heating our buildings (0.7 t). The biggest part of our footprint is from consumables and equipment (41 %) (7 t). Since our institute is still growing, adding extra lab space comprises 14 % of our ${\mathrm{CO}}_{2\;\mathrm{eq}.}$ footprint (2.4 t).

For commuting to work for the entire year (230 d), let us assume a 20 km ride (one-way), which results in an annual 2.2 t if done by car or 0.4 t by bus.

Figure [Fig F4] summarizes these estimates and highlights that the travel-related carbon footprint is by far not negligible. Traveling to ENC from Europe, for example, emits more ${\mathrm{CO}}_{2}$ than half a year of making samples, performing NMR and computing combined.

We want to stress here that the data shown in Fig. [Fig F4] are for participation in a single conference and that the actual annual carbon footprint due to conference travel is likely to be higher for many researchers. A survey of travel behavior among scientists in Germany has found that respondents attended on average 3 conferences per year in 2019 (2.2 for PhD students, 4.8 for PIs; [Bibr bib1.bibx16]).

Considering that conducting experiments is the core of our profession and the prerequisite for presenting data at a conference, it seems evident that traveling is a very significant factor in the ${\mathrm{CO}}_{2}$ emissions of researchers and may be one factor that could be reduced without significantly impacting scientific output.

## 4 Strategies to reduce conference carbon footprint

What can we do as a community and as individuals to reduce the carbon footprint from conference travel? We believe there are several avenues, which range from “technical” solutions (e.g., where to host a conference) to more “mindset” approaches.

**Figure 5 F5:**
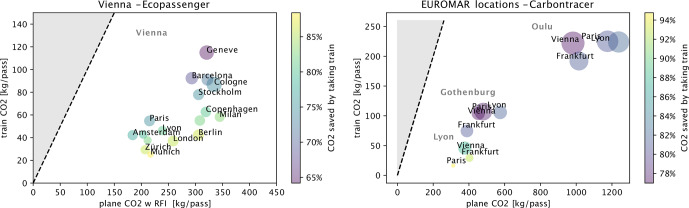
Assessing the carbon footprint of train and plane travel for a set of chosen destinations (Oulu, Gothenburg, Lyon) and cities of origin. The x ( y ) coordinate of each point indicates how much ${\mathrm{CO}}_{2}$ is emitted on average per passenger by flight (train). The color of each point shows the fraction of ${\mathrm{CO}}_{2}$ saved by taking a train instead of a flight, and the gray area of the plot corresponds to cases when it is more ecological to take a flight. Finally, the size of the dot is proportional to the ratio of estimated travel time by train and plane between the respective cities, ranging from 0.67 for Lyon ⟷ Paris to 8.3 for Oulu ⟷ Vienna.

### 4.1 Comparing train and plane travel

One possibility for reducing the carbon footprint is to choose the mode of transport wisely. To establish a solid quantitative basis for comparing trains and planes, we have conducted a review of the relevant literature. To explicitly avoid any greenwashing of trains, we have considered not only the energy required for the transport itself (direct emissions), but also emissions related to building and maintaining the infrastructure. Moreover, we have explicitly considered the energy mix of the electricity grid (country-dependent), the actually traveled distance (which is most often longer by train than by plane as the trajectory is bound to the railway grid), the infrastructure-related emissions for construction, maintenance and operation (railway network, buildings, etc.), and typical passenger occupation of trains (including night trains) and planes. Various sets of assumptions, along with model calculations based on these assumptions, are shown in the Appendix. Figure [Fig F5] shows estimated ${\mathrm{CO}}_{2\;\mathrm{eq}.}$ emissions of train and plane travel for a set of cities in Europe. As examples, we have chosen journeys from a few European cities (Frankfurt, Vienna, Paris, Lyon) to the upcoming locations (at the time of writing) of EUROMAR (Oulu, Gothenburg, Lyon). In the case of Oulu, we have also accounted for the ferry transport between Stockholm and Turku. For all these cases, train travel emits much less ${\mathrm{CO}}_{2\;\mathrm{eq}.}$ . The reductions range from about 90 %–95 % (i.e., a 10–20-fold reduction) to about 75 % (4-fold reduction). We note here that for air travel, we have assumed direct flights; stopovers add around 100 kg ${\mathrm{CO}}_{2}$ for an additional takeoff [Bibr bib1.bibx7].

In our estimations, we varied parameters such as the radiative forcing index (RFI) as well as the sources of direct emissions estimations (explained in detail in the Appendix). Figure [Fig F5] shows a representative and intermediate result of our analyses. To demonstrate the range of estimates we got by varying the RFI and other parameters, we present the “worst-case” and “best-case” scenarios in Fig. S2. In the “worst-case” scenario, taking a train “only” saves 40 %–80 % of ${\mathrm{CO}}_{2}$ , while in the “best-case” scenario, taking a train instead of a plane saves up to 95 % of emissions.

These data demonstrate that at the individual traveler's level, the choice of transport is a meaningful way to reduce the carbon footprint. However, for longer distances, this is often not a viable option. Moreover, the emission reduction also tends to decrease for longer distances traveled, in part because train trajectories are longer than those of direct flights. We also note that, partly due to political choices, such as the tax exemption of kerosene, train travel tends to be more expensive and may therefore not be possible for this reason.

**Figure 6 F6:**
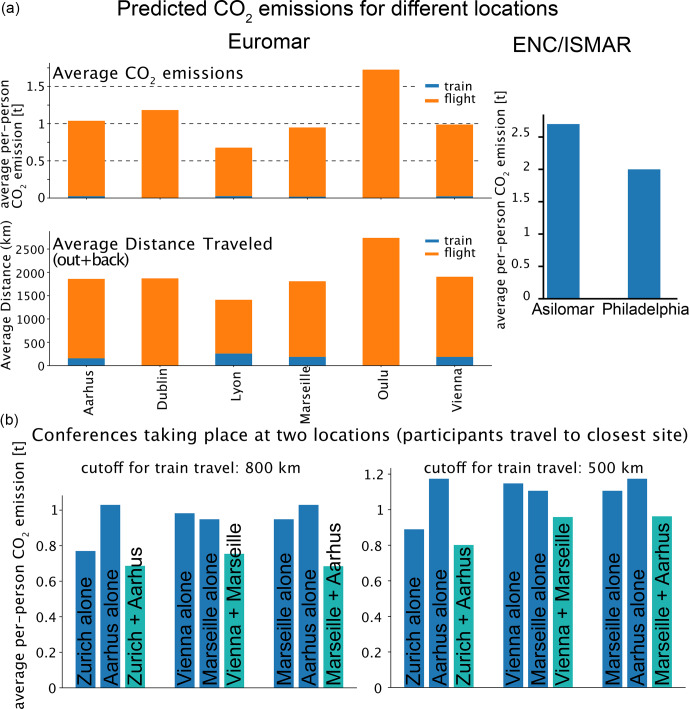
Possible strategies to reduce conference-related carbon emissions. **(A)** Prediction of ${\mathrm{CO}}_{2}$ emissions for various EUROMAR conference locations. To generate these predictions, we used the list of attendees of the 2024 EUROMAR (removing all but “local” participants from Bilbao). The distances of all attendees to the cities shown here were calculated with *Carbontracer* and converted to ${\mathrm{CO}}_{2\;\mathrm{eq}.}$ emissions. The lower panel shows the distances traveled. As in Fig. [Fig F3], participants were assumed to use train travel if their distance was <800 km. As a control, the Aarhus conference was predicted based on our assumption, and it matches reasonably well the one calculated from the actual Aarhus list. The panel on the right shows a calculation of ENC, using the participant list for ENC 2025 and performing the calculations as if it had taken place at the East Coast (Philadelphia). **(B)** Model calculations for decentralized conferences with two simultaneous locations. The attendee list from EUROMAR 2024 (as in **A**) was used to predict the emissions if the conference had taken place in Zürich, Aarhus, Marseille or Vienna (dark blue) and if it had taken place in a joint manner, whereby each participant travels to the conference site closer to their home institution. We assumed that participants would choose train travel up to 800 km (left) or 500 km (right).

### 4.2 The choice of the conference location

The significant variability in the per-person emissions of previous conferences (Fig. [Fig F3]) reveals that the location of the conference influences carbon emissions. Naturally, a more central location not only shortens the cumulative distance traveled but also increases the share of train travel. Based on data from previous EUROMAR conferences, we set out to predict the average carbon emissions per person for different conference locations (Fig. [Fig F6]A). Choosing a central conference site, such as Frankfurt, Lyon or Vienna, can reduce the per-attendee emissions by a factor in excess of 2, compared to more remote locations like Northern Scandinavia or Israel. Flight distances to central conference locations are shorter and train travel is feasible for a larger number of participants. For a conference the size of EUROMAR, this means a reduction of approximately 500–800 t of ${\mathrm{CO}}_{2}$ . Of course, besides the ${\mathrm{CO}}_{2}$ savings, it should be noted that colleagues working in places further away from the “center of mass” of the distribution are disadvantaged by systematically choosing more central locations, a factor that shall be considered.

We performed a model calculation for ENC, using the participants from the 2025 edition (which took place in California), and calculated the emission if the conference had taken place on the East Coast of the USA. We predict a ca. 25 % saving (400–500 t ${\mathrm{CO}}_{2}$ overall), which is due to the shorter distance for Europeans and possibly the density of NMR groups on the East Coast.

### 4.3 Multiple parallel virtually connected conference sites

A possible solution to reduce the carbon footprint of conferences is to decentralize them by organizing events at several local hubs, which are connected virtually. Such a conference would then have several parallel sessions – which is common to EUROMAR, ENC and ICMRBS anyhow – and attendees would choose to listen to a talk happening locally or being streamed. A possible modality, presented previously [Bibr bib1.bibx31], may look as follows. Initially, the organizers determine the event dates, select a primary location and identify several optional secondary sites. Next, they publicly announce the conference and main location and begin accepting participant applications. Then, taking into account the number and geographic distribution of applicants, participants are allocated to a final selection of venues in a way that minimizes total carbon emissions and maintains suitable attendance levels at each site. Finally, sessions proceed independently at each location, except during key plenary events, which are shared across all venues via videoconferencing. A case study of an international conference predicted this approach would cut emissions by one-third with three conference sites (one each in Japan, Europe and the USA) [Bibr bib1.bibx31]. [Bibr bib1.bibx25] present a similar strategy of dividing a conference into three hubs and connecting individual venues virtually, which could, according to their estimates, save up to 80 % of GHG emissions for a US-held conference.

We have performed a model calculation for a split conference to evaluate the potential reduction in carbon footprint. Figure [Fig F6]B shows several model calculations for three possible parallel locations. Savings of the order of 25 % appear realistic, assuming that participants choose the closest hub. This number is similar to the one estimated in the study by [Bibr bib1.bibx31].

### 4.4 Online-only conference

Online-only conferences have a much lower carbon footprint. Carbon emissions of online meetings include the participants' devices, the internet infrastructure and the data centers. For example, Zoom/Google Meet/Teams video conferencing is estimated to result in ≈0.150 to 0.250 kg ${\mathrm{CO}}_{2\;\mathrm{eq}.}$ h^−1^ per participant, which for an 8 h meeting per day amounts to 1.6 kg ${\mathrm{CO}}_{2\;\mathrm{eq}.}$ per person. This number is 2 to 3 orders of magnitude smaller than what we calculated for in-person meetings. A study of a large astronomy conference, for example, concluded that the online-only version reduced the carbon footprint by a factor of 3000; [Bibr bib1.bibx22] reported a 200-fold reduction for European political science conferences. Clearly, online-only meetings would result in a dramatic reduction in carbon emissions of MR meetings but could potentially be just as productive for advancing science and collaboration. One could argue that if an on-site conference produces 200 times more ${\mathrm{CO}}_{2}$ , it should have at least 200 times better scientific output than an online conference. How exactly to measure the scientific outcome of a conference and how to weigh it against the GHG emissions are to be determined in future research. Reasonable metrics could be used to navigate the decision-making of conference formats.

Online conferences also have advantages: they can lift barriers to attendance for more junior scientists, scientists geographically distant from the “hubs” of research institutions and conference venues, and scientists at institutions with limited funding as well as parents and caregivers in general [Bibr bib1.bibx19]. Indeed, during the pandemic, for example, the attendance at the largest European meeting for geoscience rose by over 60 % [Bibr bib1.bibx25]. More affordable online formats may thus not only reduce carbon footprint by a factor of hundreds to thousands, but also bring us closer to equal opportunities for all groups of scientists.

We argue that an online format can actually be particularly useful for poster presentations. In-person poster sessions often suffer from noisy and crowded locations; often poster presenters wait for “clients”, presenters are not around or the posters are displayed only during part of the conference. Besides, printing and transporting posters is often cumbersome. Online formats, such as those explored by the Global NMR Discussion Meetings (https://www.globalnmr.org/online-conference/, last access: 15 May 2025), may provide a better experience.

### 4.5 Less frequent conferences, joint meetings

An obvious way to reduce carbon emissions is to have fewer conferences and/or limit the number of participants per conference or, more precisely, the total distance traveled by participants for all the conferences they attend. Without compromising quality too much, one may achieve this by having meetings back-to-back at the same location. For example, a EUROMAR conference may be preceded by a more specific conference on small molecule NMR, for instance. This concept already exists in the form of satellite meetings that often take place before/after EUROMAR meetings. Similarly, we believe that attending a conference for its full duration – rather than leaving early, possibly to travel to the next meeting – is a meaningful way to improve the benefit-to-footprint ratio. Likewise, holding meetings biannually rather than annually may decrease the emissions by up to 50 %, as [Bibr bib1.bibx25] point out, or alternating between in-person and online editions.

### 4.6 Embracing more local meetings with fewer long-distance invitees

We believe that it is often seen as a mark of success for a meeting to have as wide a geographical distribution of attendees as possible. In light of the need to reduce the carbon footprint, this view shall be reconsidered to adopt a climate-conscious mindset that conferences are best attended mostly by local scientists (local meaning within a country or a continent, which is not that local really), mixed with a small number of international scientists to foster and maintain cross-continental exchange.

A scenario along these lines with the highest reduction in GHG emissions would suggest that attendees who would otherwise need to travel long distances by plane could join online, while those able to travel with a low ${\mathrm{CO}}_{2}$ footprint by train would attend in person. Since a small number of long-haul flights often account for a disproportionately large share of total emissions, as previous work has shown [Bibr bib1.bibx25], this strategy could substantially reduce the overall carbon footprint of conferences. Importantly, the experience for most attendees would remain similar to that of a traditional in-person meeting, as the majority could still gather on-site.

### Reassessing the benefit of conference attendance from a career perspective

On a personal level, choosing to attend fewer conferences is one of the most direct ways to reduce one's carbon footprint. Such a choice, of course, comes with a careful evaluation of the benefits of attending a conference. Legitimate science-based reasons for attending conferences include staying up-to-date with the latest developments, networking with researchers and vendors, initiating collaborations, and increasing professional visibility. These goals appear especially important for early-career researchers seeking academic positions. For example, in a survey of doctoral students and postdocs in Germany, respondents indicated that conference travel had contributed to collaborative projects and publications. Notably, 8 % of postdocs reported having received a job offer as a result [Bibr bib1.bibx17]. Notwithstanding the potential benefits, studies show that beyond a certain threshold, increased travel does not correlate with higher academic performance [Bibr bib1.bibx42]. This study examined air travel emissions alongside scholarly metrics, such as the h index, and found no significant relationship between the volume of air travel and research output. This finding implies that while some conference participation can be professionally beneficial, excessive travel does not translate into greater academic success.

## 5 Conclusions

In light of the climate crisis, our society will need to take swift action and decide how to restructure many aspects of our lives. Climate research, as well as the increasing number of extreme weather events, shows that change is not only required but inevitable. This topic is sensitive, and we all have opinions on the importance of conferences and their modalities, as well as the usefulness and necessity of measures to reduce the carbon footprint. Approaching this topic in a spirit of respect and open-mindedness is undoubtedly crucial. We hope to have provided valuable resources for the community and for each individual to make informed decisions and initiate a discussion on potential changes within the MR community, all while recognizing the importance of meeting to exchange ideas and advance the field together. Studies like the present one will benefit from more targeted data: in particular, it will be useful to directly ask participants/the MR community about their travel habits, preferences and willingness to reduce the community's carbon footprint.

## Supplement

10.5194/mr-6-243-2025-supplement The supplement related to this article is available online at https://doi.org/10.5194/mr-6-243-2025-supplement.

## Data Availability

The data shown in this paper were collected from abstract books, which are publicly available for EUROMAR 2017, 2018, 2019, 2022 and 2023, and lists of participants, which we either got from the organizers or downloaded via the conference app at conferences that Paul Schanda attended. These lists of attendees' affiliations (in anonymized form) as well as the scripts to convert these lists to distances and then to ${\mathrm{CO}}_{2\;\mathrm{eq}.}$ amounts are available with ISTA's repository, Research Explorer, which fulfills the FAIR criteria, with the accession number 20242, entitled “Data of: Quantifying the carbon footprint of conference travel: the case of NMR meetings”. The data are available free of charge at 10.15479/AT-ISTA-20242 [Bibr bib1.bibx39].

## Appendix A Conference travel data used in this analysis

The analysis of past conferences was based on lists of participants which we obtained either (1) directly from organizers as a compiled anonymized spreadsheet of affiliations, (2) as a list available to participants via a conference app or (3) as abstract books available from the conference's website. For the latter case, we combined an automatic text extraction tool written in-house in the Python programming language using a Google API with manual curation. We then performed (largely manual) internet searches to associate the participants' affiliations with the city where their institute is located. We assumed that the participant traveled from this city to the conference site. A Python script that calls the *Carbontracer* API was written to convert start and end points of the trips to distances (explicitly taking into account the actual rail or flight distance), as well as the emissions related to the direct transport. We have included indirect emissions resulting from infrastructure construction and maintenance, as shown in the following sections.

## Appendix B Estimating the carbon footprint of different modes of transport

### B1 The carbon footprint of train travel

We estimate the carbon footprint of train travel by adding up the *direct emissions* $D^{\left(t\right)}$ of the train journey and the *indirect emissions* $I^{\left(t\right)}$ associated with construction and maintenance of the railway infrastructure and emissions resulting from heating and cooling of the station buildings. The index in the exponent of individual variables indicates the corresponding mode of transport; here ^(t)^ stands for train, and later ^(p)^ indicates plane and ^(f)^ represents ferry.

While tools exist for calculating the direct emissions (i.e., the production of energy for propelling the trains), including the different carbon intensities of electricity production in different countries (see Sect. B4), we wanted to obtain a reliable estimation of $I^{\left(t\right)}$ . To do that, we use the findings of [Bibr bib1.bibx26], who estimate the emissions associated with the construction and maintenance of the Austrian railway network to be ${\text{CM}}^{\left(t\right)}=234\;730$ t of ${\mathrm{CO}}_{2\;\mathrm{eq}.}$ . We further use information from the ÖBB report [Bibr bib1.bibx32], which states that the yearly emissions associated with heating and cooling of the station buildings are around $B^{\left(t\right)}=49\;500$ t of ${\mathrm{CO}}_{2\;\mathrm{eq}.}$ in 2021 – a number comparable to the total direct emissions of passenger traffic that year [Bibr bib1.bibx32].

Further, according to ÖBB [Bibr bib1.bibx33], the number of passenger kilometers (pkm) traveled in 2022 was $T=11.4\times 10^{9}$ pkm. We take the estimate from 2022 to avoid the effects of decreased travel due to the Covid-19 pandemic.

Finally, we compute the mean indirect emissions per passenger kilometer:

B1
$$\gamma =\frac{{\text{CM}}^{\left(t\right)}+B^{\left(t\right)}}{T}=24.9\;g\;{\mathrm{CO}}_{2\;\mathrm{eq}.}\;{\mathrm{pkm}}^{-1}.$$

The above estimate of γ is consistent with previously reported estimates of the carbon cost of railway infrastructure (Fig. 5.4; [Bibr bib1.bibx40]) for several European countries of the order of 8–20 g ${\mathrm{CO}}_{2\;\mathrm{eq}.}$ pkm^−1^. Our estimate is on the higher end and thus conservative when assessing the ecological benefits of train travel.

To generalize our calculations for travel outside Austria, we assume that the construction and maintenance of a kilometer of rails have a similar carbon footprint in Western European and Central European countries. The reasoning is as follows: first, the rail materials used are the same, and their source is often the same across European countries, and thus they are associated with similar emissions. Second, [Bibr bib1.bibx26] conclude that a substantial amount of the carbon footprint of maintenance originates from the use of diesel engine vehicles, whose footprint is also region-independent. We also assume that the amount of ${\mathrm{CO}}_{2\;\mathrm{eq}.}$ emitted per pkm for operating station buildings is, on average, similar across European countries.

We calculate the total emission $E_{\mathrm{AB}}^{\left(t\right)}$ for a train travel of one passenger from city A to city B as the sum of direct $D_{\mathrm{AB}}^{\left(t\right)}$ and indirect $I_{\mathrm{AB}}^{\left(t\right)}$ emission. $l_{\mathrm{AB}}^{\left(t\right)}$ is the distance covered by the train between cities A and B.

B2
$$E_{\mathrm{AB}}^{\left(t\right)}=D_{\mathrm{AB}}^{\left(t\right)}+I_{\mathrm{AB}}^{\left(t\right)}=D_{\mathrm{AB}}^{\left(t\right)}+\gamma l_{\mathrm{AB}}^{\left(t\right)}$$

### B2 The carbon footprint of air travel

Like train travel, flight emissions consist of direct and indirect emissions. Indirect emissions comprise airport infrastructure as well as the RFI, the key emission factor in air travel. The RFI indicates how much more potent greenhouse gases emitted at a certain altitude are compared to emissions on the ground. Depending on the altitude of plane travel, the RFI ranges from 1.3 to 3 [Bibr bib1.bibx28]. The indirect emissions associated with the construction and maintenance of airport buildings are estimated to be around $B^{\left(p\right)}=15$ % of the aviation footprint [Bibr bib1.bibx15]. The great-circle distance – the shortest distance connecting points A and B on a sphere – is usually used to calculate the distance of flights. It does not accurately represent real-world flights as it does not account for start and landing phases, indirect flight routes, delays, or waiting time in the air. Hence, the great-circle distance has to be adjusted by an uplift factor of 8 % for compensation [Bibr bib1.bibx9]. The formula for estimating flight emissions per passenger between cities A and B is

B3
$$E_{\mathrm{AB}}^{\left(p\right)}=D_{\mathrm{AB}}^{\left(p\right)}(\text{RFI}+B^{\left(p\right)}),$$

where $D_{\mathrm{AB}}^{\left(p\right)}$ is the direct flight emission per passenger calculated from the estimated flight distance corrected for the uplift factor.

### B3 The carbon footprint of ferry travel

Similar to travel by land and air, the emissions of ferry travel comprise direct and indirect emissions. The direct emissions range from 40 to 300 g ${\mathrm{CO}}_{2\;\mathrm{eq}.}$ pkm^−1^ [Bibr bib1.bibx27]. 40 g ${\mathrm{CO}}_{2\;\mathrm{eq}.}$ pkm^−1^ appears to be the most reasonable number as it allocates emissions between freight and passengers in a weight-dependent manner [Bibr bib1.bibx27]. Nevertheless, we used $D_{\mathrm{pkm}}^{\left(f\right)}=0.2$ kg ${\mathrm{CO}}_{2\;\mathrm{eq}.}$ pkm^−1^ in our exemplary calculations to stay conservative for our claim that any other transport is more favorable in ${\mathrm{CO}}_{2}$ emissions than flying. We assumed that ports are the main contributors to indirect ferry emissions. The greenhouse gas (GHG) emissions at the port of Stockholm were 523 t ${\mathrm{CO}}_{2\;\mathrm{eq}.}$ in 2024 [Bibr bib1.bibx34], when it served as a hub for 7.2 million passengers. This leads to indirect emissions of 0.07 kg ${\mathrm{CO}}_{2\;\mathrm{eq}.}$ per passenger. Assuming that ports emit the same level of emissions across Europe and that passengers always use a departure and an arrival port, this results in $I^{\left(f\right)}=0.14$ kg ${\mathrm{CO}}_{2\;\mathrm{eq}.}$ per passenger of indirect emissions per ferry journey. The equation for calculating ferry emissions is the following:

B4
$$E_{\mathrm{AB}}^{\left(f\right)}=D_{\mathrm{pkm}}^{\left(f\right)}l_{\mathrm{AB}}^{\left(f\right)}+I^{\left(f\right)},$$

where $l_{\mathrm{AB}}^{\left(f\right)}$ is the distance covered by the ferry between cities A and B.

### B4 Calculating GHG emissions of different ways of traveling

As a basis for estimating the total GHG emissions of different ways of traveling, we used web tools that calculate direct emissions D caused by a specific trip. To get a range of emission estimates, we evaluate the carbon footprint using multiple sources of information and multiple alternative settings. We take or calculate D directly from two available online tools: *Ecopassenger* [Bibr bib1.bibx21] and *Carbontracer* [Bibr bib1.bibx41].

*Ecopassenger* considers GHG emissions that are directly caused by operating the vehicles and the final energy consumption. For railway travel, the route length is determined by the polygon defined by all train stops on the way to the destination. The route length between stops is based on the line of sight extended by 20 % to 30 %, depending on the case. *Ecopassenger* estimates GHG emissions by considering the average national electricity mix of the countries traveled. It allows the selection of either the “National production electricity mix” or “Railways mix” and evaluates both scenarios. To estimate the total infrastructure including GHG emissions, the resulting direct emissions $D_{\left(\mathrm{AB}\right)}^{\left(t\right)}$ need to be extended as displayed in Eq. (B2).

Flight route lengths are calculated based on the great-circle distance, which is corrected for additional distance such as wait loops by adding 50 km. Since planes travel longer at higher altitudes when covering longer distances, flights are corrected with RFI factors of between 1.26 for distances up to 500 km and 2.5 for distances above 1000 km, but *Ecopassenger* also shows the value without RFI.

The *Ecopassenger* platform returns the plane travel emissions $P_{\mathrm{AB}}^{\left(p\right)}$ per passenger for the trip between cities A and B. To obtain the estimate of total emissions $E_{\mathrm{AB}}^{\left(p\right)}$ , we add the infrastructure factor $B^{\left(p\right)}=15\;\%$ . Since the *Ecopassenger* emissions estimate already includes RFI, the resulting equation for $E_{\mathrm{AB}}^{\left(p\right)}$ is as follows:

B5
$$E_{\mathrm{AB}}^{\left(p\right)}=P_{\mathrm{AB}}^{\left(p\right)}+\frac{P_{\mathrm{AB}}^{\left(p\right)}}{\text{RFI}}B^{\left(p\right)},$$

where, based on Eq. ([Disp-formula App1.Ch1.S2.E3]), the direct flight emissions per passenger are $D_{\mathrm{AB}}^{\left(p\right)}=\frac{P_{\mathrm{AB}}^{\left(p\right)}}{\text{RFI}}$ and the indirect emissions $I_{\mathrm{AB}}^{\left(p\right)}=D_{\mathrm{AB}}^{\left(p\right)}\left(\text{RFI}-1+B^{\left(p\right)}\right)$ .

*Carbontracer* is based on life cycle analysis (LCA) GHG emissions per person per kilometer of the different vehicles. For all types of travel, it includes not only direct emissions, but also the construction, maintenance and disposal of the respective vehicle. Due to the use of various routing maps, the actual train routes are calculated. *Carbontracer* considers the national electricity mixes for train travel and displays GHG emissions per person when traveling seated, in a couchette or in a sleeping car. To estimate the total GHG emissions, the output given by *Carbontracer* needs to be plugged into Eq. (B2).

Flight routes are calculated using another set of routing maps and compensated by application of the already-mentioned uplift factor of 8 %. *Carbontracer* independently of flight distance always applies an RFI of 2.

To include airport infrastructure emissions, the flight emissions according to the *Carbontracer* platform $P_{\mathrm{AB}}^{\left(p\right)}$ need to be corrected as follows:

B6
$$E_{\mathrm{AB}}^{\left(p\right)}=P_{\mathrm{AB}}^{\left(p\right)}+\frac{P_{\mathrm{AB}}^{\left(p\right)}}{2}B^{\left(p\right)}.$$

The equation takes the same form as Eq. ([Disp-formula App1.Ch1.S2.E5]) with RFI fixed at 2 for *Carbontracer*, thus the 2 in the denominator. Then, analogously to the *Ecopassenger* section above, the direct flight emissions per passenger are $D_{\mathrm{AB}}^{\left(p\right)}=\frac{P_{\mathrm{AB}}^{\left(p\right)}}{2}$ and the indirect emissions $I_{\mathrm{AB}}^{\left(p\right)}=D_{\mathrm{AB}}^{\left(p\right)}\left(2-1+B^{\left(p\right)}\right)$ .

Figure S2 shows the comparisons of GHG emissions for plane vs. train travel between Vienna and several major European cities that are common destinations for business travel of ISTA employees. The color of each point signifies the fraction of ${\mathrm{CO}}_{2}$ saved by taking a train. We tested scenarios based on multiple assumptions as follows:

- The direct emissions estimates are obtained from two distinct platforms *Carbontracer* and *Ecopassenger*.
- The RFI value ranges between 1.3 and 2.7.
- The electricity mix used by the railways is the “national” or “railway” mix.
- The train is a day train or night train.

Plots in Fig. S2A show 3 of the most adverse scenarios for trains, where taking a train saves the least amount of ${\mathrm{CO}}_{2\;\mathrm{eq}.}$ . It includes cases with low RFI and traveling by night train, which reduces the train's capacity and thus increases the emissions per passenger. Even in those scenarios, a train saves at least 40 % ${\mathrm{CO}}_{2\;\mathrm{eq}.}$ compared to air travel. Figure S2B shows the representative case scenario, where an intermediate fraction of ${\mathrm{CO}}_{2\;\mathrm{eq}.}$ is saved by taking a train instead of a plane. This is the scenario where the RFI is altitude-dependent. Typically, a journey by train saves around 75 % of ${\mathrm{CO}}_{2\;\mathrm{eq}.}$ . Figure S2C shows the most favorable scenario for trains when RFI is assumed to be high and railways are assumed to run on a greener electricity mix than the national electricity mix in the respective countries. In such a case, up to 95 % of GHG emissions can be saved by avoiding a flight.

Finally, in Fig. S2D we show the distribution of the effective factor ξ that is defined as the ratio of total GHG emissions of a train journey and the direct emissions associated with moving the train itself.

B7
$$\xi_{\mathrm{AB}}=\frac{D_{\mathrm{AB}}^{\left(t\right)}+I_{\mathrm{AB}}^{\left(t\right)}}{D_{\mathrm{AB}}^{\left(t\right)}}$$

The average value of $\bar{\xi }\approx 2.5$ tells us, as a rule of thumb, by how much one should multiply the GHG emissions estimate by platforms such as *Ecopassenger* to get a realistic ${\mathrm{CO}}_{2}$ estimate including the costs of railway infrastructure.

ξ ranges from just above 1 for night trains and journeys to countries with the least clean electricity mix, where the indirect emissions are not larger than the direct emissions and from up to 7 for Austria, where trains are powered exclusively by hydropower, and therefore the indirect emissions comprise the majority of the total emissions. The estimate for Austria based on data by [Bibr bib1.bibx26] lands at 6.9.
