# Supplementary material for: Quantifying the carbon footprint of conference travel: the case of NMR meetings
Source: Magn Reson (Gott). 2025 Nov 10;6(2):243–56. doi: 10.5194/mr-6-243-2025 (PMC12658769; doi:10.5194/mr-6-243-2025)
Supplement: The supplement related to this article is available online at https://doi.org/10.5194/mr-6-243-2025-supplement. [file mr-6-243-2025-supplement.pdf]

Supplement of Magn. Reson., 6, 243–256, 2025
https://doi.org/10.5194/mr-6-243-2025-supplement
© Author(s) 2025. CC BY 4.0 License.

Supplement of

Quantifying the carbon footprint of conference travel: the case of NMR meetings

Lucky N. Kapoor et al.

Correspondence to: Paul Schanda (paul.schanda@ist.ac.at)

The copyright of individual parts of the supplement might differ from the article licence.

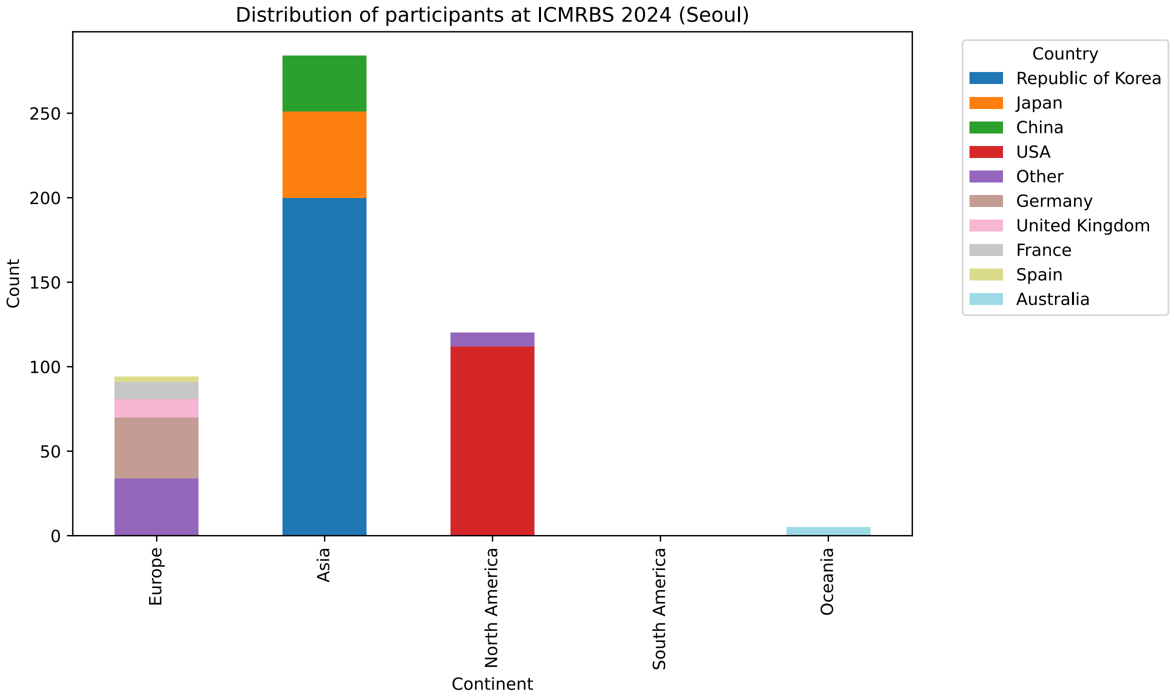

Figure S1. Distribution of participants at the 2024 ICMRBS in Seoul.

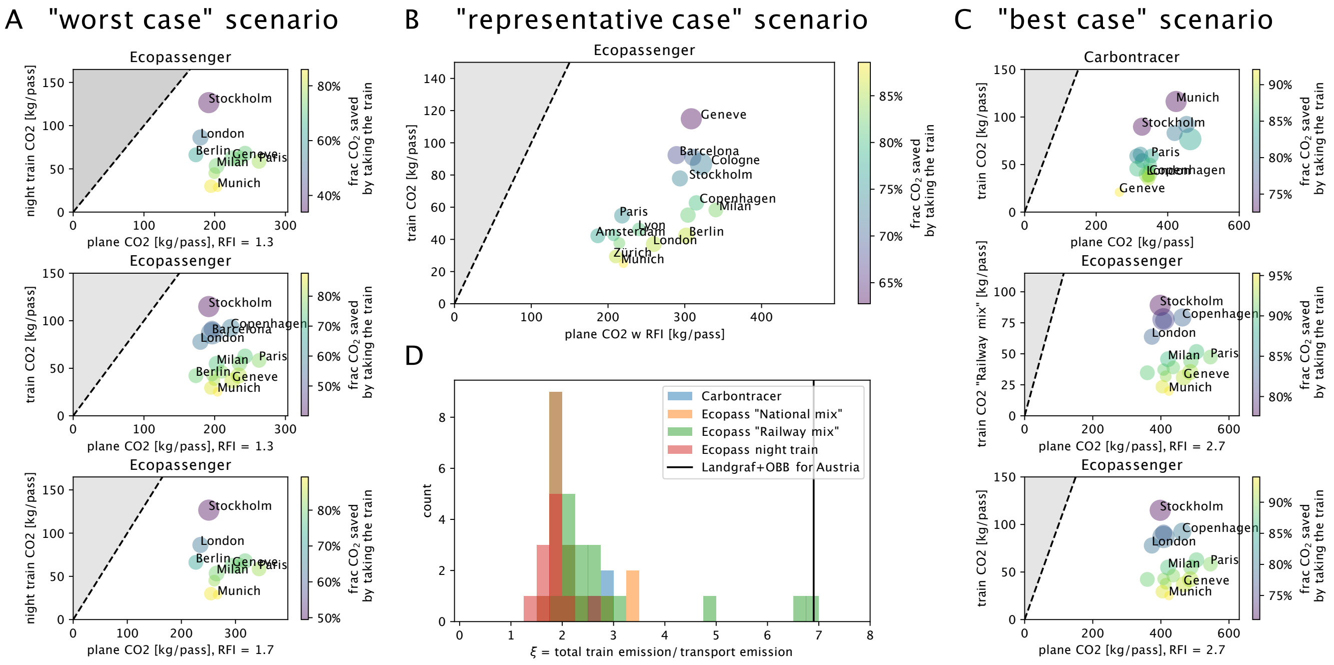

Figure S2. Analysis of CO_2 footprint of trains vs planes for journeys from Vienna to major European cities. **(A-C)** The x(y) coordinate of each point indicates how much CO_2 is emitted on average per passenger by flight (train). The colour of each point shows the fraction of CO_2 saved by taking a train instead of a flight, and the grey area of the plot corresponds to cases when it is more ecological to take a flight. Furthermore, the dot size is proportional to the ratio of estimated travel time by train and plane between the respective cities. **(A)** "Worst case scenario": three alternatives when the amount of CO_2 saved by taking the train is the lowest. These arise when assuming low RFI and traveling by night train, where each passenger occupies more space. **(B)** representative case scenario where altitude-dependent RFI is considered. **(C)** Three "Best case scenarios" where taking a train saves the highest fraction of CO_2 compared to taking a plane. These are scenarios assuming high RFI and green railway electricity mix. **(D)** Distribution of ξ, the ratio of CO_2 footprint of each train journey, including emissions due to infrastructure and the CO_2 footprint of the journey itself, without infrastructure. The ratio is typically around 2-3 and serves as a "rule of thumb" for how much one should multiply the *direct* CO_2 emissions of a train journey calculated by *Ecopassenger*, *Carbontracer*, or similar platforms to get a more realistic estimate, including the footprint of infrastructure.
